# Environmental Noise-Induced Effects on Stress Hormones, Oxidative Stress, and Vascular Dysfunction: Key Factors in the Relationship between Cerebrocardiovascular and Psychological Disorders

**DOI:** 10.1155/2019/4623109

**Published:** 2019-11-11

**Authors:** Omar Hahad, Jürgen H. Prochaska, Andreas Daiber, Thomas Muenzel

**Affiliations:** ^1^Center for Cardiology-Cardiology I, University Medical Center of the Johannes Gutenberg-University Mainz, Mainz, Germany; ^2^Preventive Cardiology and Preventive Medicine, Center for Cardiology, University Medical Center of the Johannes Gutenberg-University Mainz, Mainz, Germany; ^3^German Center for Cardiovascular Research (DZHK), Partner Site Rhine-Main, Mainz, Germany

## Abstract

The role of noise as an environmental pollutant and its adverse effects on health are being increasingly recognized. Beyond its direct effects on the auditory system (e.g., hearing loss and tinnitus induced by exposure to high levels of noise), chronic low-level noise exposure causes mental stress associated with known cardiovascular complications. According to recent estimates of the World Health Organization, exposure to traffic noise is responsible for a loss of more than 1.5 million healthy life years per year in Western Europe alone, a major part being related to annoyance, cognitive impairment, and sleep disturbance. Underlying mechanisms of noise-induced mental stress are centered on increased stress hormone levels, blood pressure, and heart rate, which in turn favor the development of cerebrocardiovascular disease such as stroke, arterial hypertension, ischemic heart disease, and myocardial infarction. Furthermore, traffic noise exposure is also associated with mental health symptoms and psychological disorders such as depression and anxiety, which further increase maladaptive coping mechanisms (e.g., alcohol and tobacco use). From a molecular point of view, experimental studies suggest that traffic noise exposure can increase stress hormone levels, thereby triggering inflammatory and oxidative stress pathways by activation of the nicotinamide adenine dinucleotide phosphate oxidase, uncoupling of endothelial/neuronal nitric oxide synthase inducing endothelial and neuronal dysfunction. This review elucidates the mechanisms underlying the relationship between noise exposure and cerebrocardiovascular and psychological disorders, focusing on mental stress signaling pathways including activation of the autonomous nervous system and endocrine signaling and its association with inflammation, oxidative stress, and vascular dysfunction.

## 1. Introduction

Already in the beginning of the 20^th^ century, the Nobel prize-winning bacteriologist Robert Koch predicted that “One day man will have to fight noise as fiercely as cholera and the plague”. Indeed, of the most recent WHO Environmental Noise Guidelines for the European Region provides substantial evidence that links environmental noise exposure to adverse health outcomes [1]. According to estimations of the World Health Organization (WHO), exposure to traffic-related noise accounts for a yearly loss of more than 1.5 million years of healthy life in Western Europe with 61,000 years for ischemic heart disease, 45,000 years for cognitive impairment of children, 903,000 years for sleep disturbance, 22,000 years for tinnitus, and 654,000 years for annoyance [2]. According to conservative estimates for the European Region, exposure to noise from road traffic, railway, and aircraft leads to annoyance among 53 million and to sleep disturbance among 34 million adults, resulting each year in nearly 1.7 million additional prevalent cases of hypertension, 80,000 additional cases of hospital admissions, and to 18,000 cases of premature mortality due to ischemic heart disease and stroke [3]. A large body of epidemiological and experimental studies demonstrated that exposure to traffic noise is associated with increased risk of cerebrocardiovascular disease such as stroke, arterial hypertension, ischemic heart disease, and myocardial infarction [4, 5]. A recent meta-analysis conducted on behalf of the WHO suggested a relative risk (RR) for the incidence of ischemic heart disease of 1.08 (95% confidence interval (CI): 1.01–1.15) per 10 decibel (dB) increase in road traffic noise exposure above 50 dB based on high-quality longitudinal studies [1]. Furthermore, environmental noise exposure has been established as a phenomenon causing annoyance and mental stress reactions, resulting in sympathetic and endocrine stress reactions (i.e., increased stress hormone levels) and psychological disorders such as depression and anxiety, all of which further impair cerebrocardiovascular function [6]. Importantly, chronic noise stress may generate cerebrocardiovascular risk factors on its own by influencing hemodynamics, hemostasis, oxidative stress, inflammation, vascular function, and autonomic tone, subsequently leading to manifest cerebrocardiovascular disease [7]. The present review discusses the role of environmental noise exposure in developing cerebrocardiovascular and psychological disorders as well as their bidirectional relationship focusing on sympathetic and endocrine stress responses with subsequent onset of inflammation, oxidative stress, and vascular endothelial dysfunction.

## 2. Noise Reaction Model

**Figure 1:**
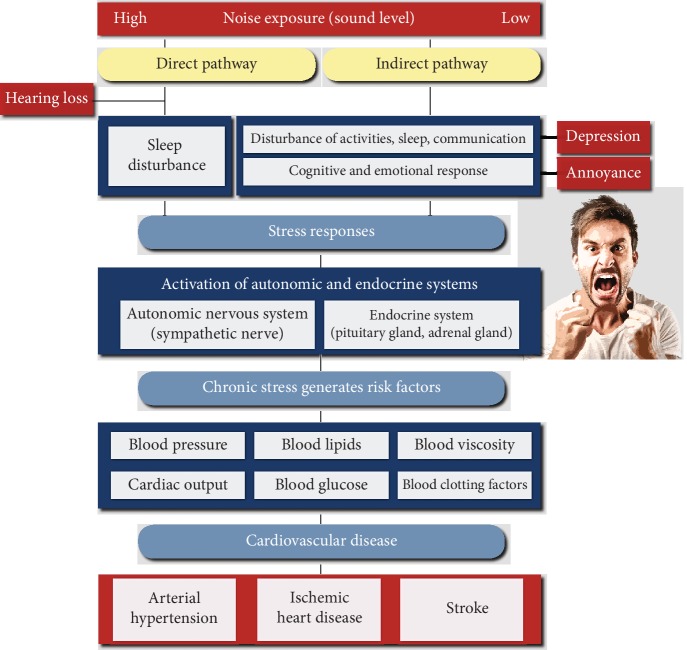
Proposed expanded noise reaction model characterizing the adverse health effects of environmental noise exposure. The direct pathway refers to effects on the auditory system by exposure to high levels of noise (e.g., hearing loss and tinnitus). The indirect pathway is associated with cognitive and emotional stress responses, leading to sympathetic and endocrine activation triggering alterations in cerebrocardiovascular risk factors. Additionally, chronic noise stress is associated with increased risk for psychological symptoms and disorders, which in turn impairs cerebrocardiovascular function. As a consequence, noise exposure may promote maladaptive coping mechanisms and decrease stress resistance, further negatively affecting cerebrocardiovascular function. Adapted from Babisch [7, 8] and Münzel et al. [5] with permission.

According to Babisch's noise reaction model, the “indirect/nonauditory pathway” (compared to the “direct/auditory pathway,” which describes effects on the auditory system by exposure to high levels of noise such as hearing loss and tinnitus) is crucial in determining adverse systemic health effects (Figure 1) [7, 8]. In this setting, low-level noise exposure interferes with communication, disturbs daily activities, and disrupts sleep, leading to sympathetic and endocrine activation and a number of cognitive and emotional reactions, including annoyance, depression, and mental stress. If the exposure persists over a period of time, the cognitive and emotional state of stress could then cause a pathophysiological cascade, resulting in increased stress hormone levels, blood pressure, and heart rate, which in turn favors the development of cerebrocardiovascular risk factors such as hypertension, arrhythmia, dyslipidemia, increased blood viscosity and blood glucose, and activation of blood clotting factors and the subsequent manifestation of cerebrocardiovascular disease such as stroke, ischemic heart disease, acute myocardial infarction, heart failure, and arterial hypertension [5, 6]. Of note, even short-term nocturnal aircraft noise exposure has been shown to be associated with takotsubo cardiomyopathy, a condition triggered by emotional stress and excessive stress hormone release (also known as broken-heart syndrome) [9]. Noise-induced annoyance has been proposed to play an intermediary role in disease development, i.e., the degree to which noise causes interference, annoyance, and mental stress may mediate the pathophysiological consequences and disease risk [10, 11]. Accordingly, traffic noise annoyance was shown to be an effect modifier or to be directly related to ischemic heart disease, hypertension, atrial fibrillation, myocardial infarction, stroke, and symptoms of cardiovascular disease [10–18].

**Figure 2:**
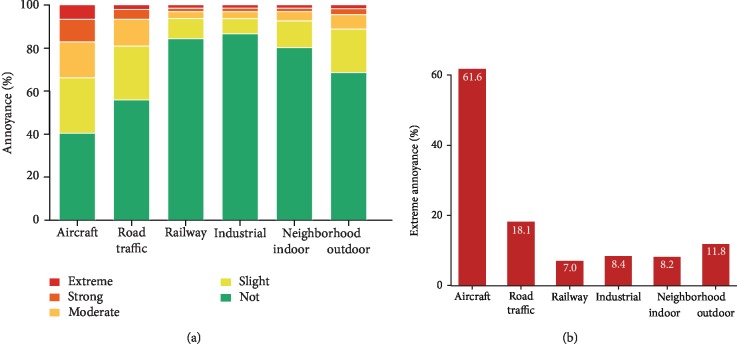
(a) Degrees of overall annoyance according to different sources of noise. (b) Sources of extreme annoyance. Adapted from Beutel et al. [19] with permission.

Additionally, considering that noise annoyance represents mental stress, it has been shown to be associated with psychological symptoms and disorders such as depression and anxiety, with the important notion that different noise sources may induce different levels of annoyance (Figure 2) [19, 20]. Importantly, chronic mental stress per se is a well-established independent risk factor for both cerebrocardiovascular and psychological disorders, while these conditions are known to negatively affect each other in a bidirectional way [21, 22]. Given this framework, chronic noise annoyance/stress may impair adaptation and increase stress vulnerability, leading to decreased stress resistance in order to cope with the stressor [15]. Instead, as a consequence of stress and onset of psychological disorders, noise exposure may promote maladaptive coping mechanisms in the manner of life style risk factors, as shown by recent studies indicating that traffic noise was related to physical inactivity, smoking, and alcohol consumption [23–25]. Thus, besides the direct adverse cerebrocardiovascular effects of noise, an indirect pathway of adverse noise effects can be assumed causing cerebrocardiovascular disease via causing psychological disorder such as depression and anxiety.

## 3. Epidemiological Evidence for Health Effects of Noise

There is extensive epidemiological evidence for the significant relationship between environmental noise exposures, in particular for exposure to noise from road traffic, railway, and aircraft, and cerebrocardiovascular as well as psychological endpoints. In the following, an overview of studies for these endpoints shall be given. This review is based on a selective search of publications in PubMed from 2005 to 2019 with focus on systematic reviews, meta-analyses, and primary studies. We used the search terms: “noise exposure” in combination with “cardiovascular disease,” “cardiovascular,” “psychological,” “psychiatric,” “depression,” and “anxiety,” following an initial rapid review and selection of the articles based on the authors' expertise. Clear descriptions of study population characteristics, adjustment for common confounders, description of inclusion and exclusion criteria, and robust methodology were at least required.

### 3.1. Ischemic Heart Disease

As mentioned, the most recent meta-analysis from 2018 by Kempen et al. found a RR of 1.08 (95% CI: 1.01–1.15) for the incidence of ischemic heart disease per 10 dB increase in road traffic noise exposure for the relationship of road traffic noise, starting as low as 50 dB [1]. Similar findings were obtained by two other meta-analyses by Vienneau et al. and by Babisch, reporting a RR of 1.06 (95% CI: 1.03–1.09) and 1.08 (95% CI: 1.04–1.13), respectively, per 10 dB/dB(A) (A-weighted decibel) increase in aircraft and/or road traffic noise exposure [26, 27]. A large population-based study from Canada showed that an increase in traffic-related noise levels per 10 dB(A) was associated with a 9% (RR: 1.09, 95% CI: 1.01–1.18) higher risk of death from ischemic heart disease [28].

### 3.2. Hypertension

A meta-analysis of 24 cross-sectional studies by van Kempen and Babisch found a 3.4% (odds ratio (OR): 1.034, 95% CI: 1.011–1.056) higher probability of prevalent hypertension per increase of 5 dB(A) in road traffic noise [29]. However, prospective studies have also indicated an association between aircraft noise exposure and incident hypertension, thereby supporting the cross-sectional findings [30, 31]. The more recent study from 2017, based on data from the large Hypertension and Exposure to Noise near Airports (HYENA) study from 6 European countries, found that an increase in nocturnal aircraft noise exposure per 10 dB was associated with an OR of incident hypertension of 2.63 (95% CI: 1.21–5.71) [31]. Findings of a Taiwanese study suggested an OR of 2.15 (95% CI: 1.08–4.26) for prevalent hypertension in subjects exposed to high levels of road traffic noise (82.2 vs. 77.2 dB(A)) [32].

### 3.3. Myocardial Infarction

Three Scandinavian studies have reported on the association between road traffic noise exposure and myocardial infarction [33–35]. In a Swedish case-control study, an OR of 1.38 (95% CI: 1.11–1.71) was found for noise levels of <50 vs. ≥50 dB(A) after exclusion of subjects with hearing loss or exposure to noise from other sources [33]. Similar results were obtained in two large Danish cohorts, indicating a hazard ratio (HR) of 1.12 (95% CI: 1.03–1.21) per interquartile range increase in noise levels and an incidence rate ratio (IRR) of 1.12 (95% CI: 1.02–1.22) per increase of 10 dB, respectively [34, 35]. Concerning risk of death from cardiac causes, a nationwide study from Switzerland including more than 4 million individuals analyzed the association between traffic noise exposure and mortality due to myocardial infarction [36]. The results indicated a 3.8% (HR: 1.038, 95% CI: 1.019–1.058) higher risk for road traffic noise, a 2.6% (HR: 1.026, 95% CI: 1.004–1.048) higher risk for aircraft noise, and a 1.8% (HR: 1.018, 95% CI: 1.004–1.031) higher risk for railway noise per 10 dB increase in noise levels. In a Canadian study of nearly 28,000 lumber mill workers, an increased risk of fatal myocardial infarction in relation to occupational noise exposure was observed (RR: 1.05, 95% CI: 1.1–2.2 for ≥115 vs. <100 dB(A)) [37]. A German case-control study, which was based on secondary data of the Noise-Related Annoyance, Cognition, and Health (NORAH) project, using data of more than 1 million individuals living in the Rhine-Main region, showed that road traffic and railway noise exposure increased risk of myocardial infarction by 2.8% (OR: 1.028, 95% CI: 1.012–1.045) and by 2.3% (OR: 1.023, 95% CI: 1.005–1.042) per 10 dB increase, respectively, whereas the association was weaker for aircraft noise (OR: 0.993, 95% CI: 0.966–1.020) [38].

### 3.4. Stroke

Sorensen et al. found road traffic noise to increase the risk of hospitalization due to incident stroke by 14% (IRR: 1.14, 95% CI: 1.03–1.25) [39]. A large population-based study including 3.6 million individuals living around Heathrow airport in London showed that aircraft noise exposure during the day as well as at night was associated with increased risk of hospitalization due to incident stroke [40]. A RR of 1.29 (95% CI: 1.14–1.46 for >55 vs. ≤50 dB) was determined for nocturnal aircraft noise exposure, whereas a RR of 1.24 (95% CI: 1.08–1.43 for >63 vs. ≤51 dB) was estimated for daytime exposure. Comparable results were reported for stroke-related mortality in this study. A further study, likewise conducted in London including 8.6 million individuals living around, revealed road traffic noise exposure to be associated with an elevated risk of stroke-related hospitalization during the day (RR: 1.05, 95% CI: 1.02–1.09 for >60 vs. <55 dB in individuals aged ≥25 to 74 years) and at night (RR: 1.05, 95% CI: 1.01–1.09 for 55-60 vs. <55 dB in individuals aged ≥75 years) [41]. Results from a NORAH case-control study revealed that stroke risk was increased by 1.7% (OR: 1.017, 95% CI: 1.003–1.032) for road traffic noise and by 1.8% (OR: 1.018, 95% CI: 1.001–1.034) for railway noise per 10 dB increase [42]. This association was weaker in case of aircraft noise (OR: 0.976, 95% CI: 0.953–1.000).

### 3.5. Other Endpoints

Another NORAH-based case-control study examined the association between exposure to traffic noise and risk of heart failure or hypertensive heart disease [43]. The study showed that increased levels of road traffic (OR: 1.024, 95% CI: 1.016–1.032), railway (OR: 1.031, 95% CI: 1.022–1.041), and aircraft noise (OR: 1.016, 95% CI: 1.003–1.030) were found to be associated with increased risk (per 10 dB increase).

**Figure 3:**
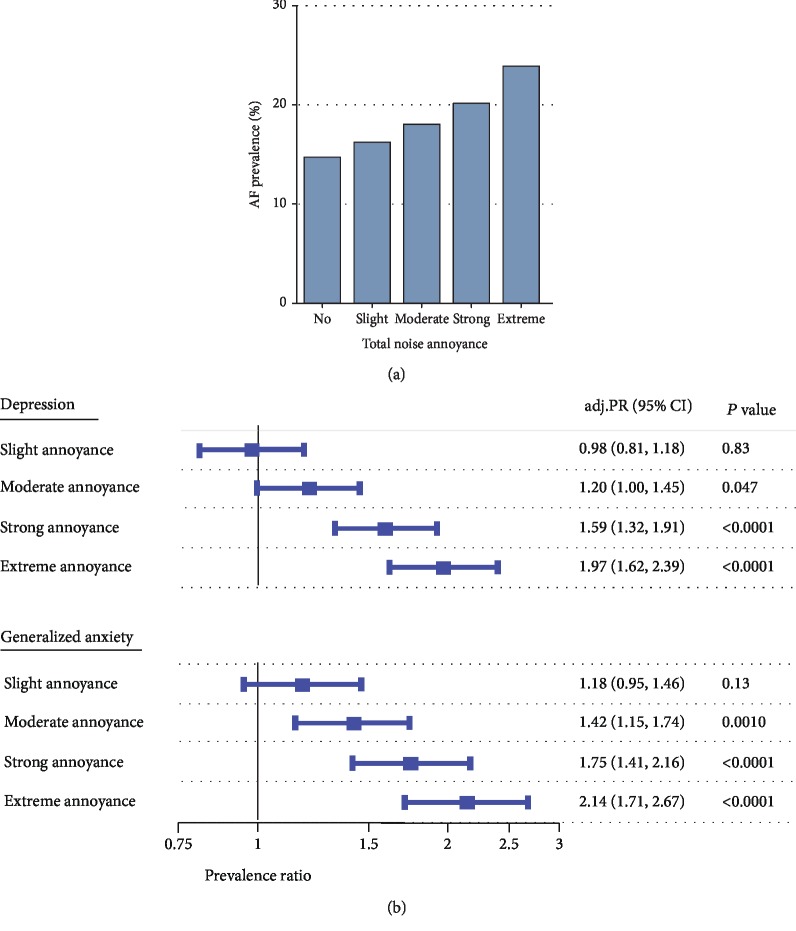
Associations between total noise annoyance, (a) atrial fibrillation, (b) depression, and generalized anxiety in the Gutenberg Health Study. AF: atrial fibrillation; adj. PR: adjusted prevalence ratio for sex, age, and socioeconomic status; CI: confidence interval. Adapted from (a) Hahad et al. [15] and (b) Beutel et al. [19] with permission.

Based on cross-sectional data from the large, population-based Gutenberg Health Study (GHS) from Germany, Hahad et al. demonstrated that annoyance to different noise sources during the day and at night, including aircraft (OR: 1.09, 95% CI: 1.05–1.13), road traffic (OR: 1.15, 95% CI: 1.08–1.22), and railway annoyance (OR: 1.13, 95% CI: 1.04–1.22, at night and per point increase in annoyance), was dose-dependently related to a higher probability of prevalent atrial fibrillation (Figure 3(a)) [15].

A Taiwanese study analyzed the association between road traffic noise exposure and physician-diagnosed cardiovascular disease [44]. An elevated OR of 2.23 (95% CI: 1.26–3.93) for prevalent cardiovascular disease per 5 dB(A) increase in noise levels was found.

A nationwide study including 6 million older people (aged ≥65 years) residing near 89 airports in the United States showed that aircraft noise exposure per 10 dB increase was associated with a 3.5% (95% CI: 0.2–7.0) higher cardiovascular hospital admission rate comprising stroke, ischemic heart disease, heart failure, arrhythmia, and peripheral vascular disease [45].

Results of the National Health and Nutrition Examination Survey (NHANES) from the United States revealed that occupational noise exposure was associated with increased risk of angina pectoris (OR: 2.91, 95% CI: 1.35–6.26 for never exposed vs. current exposed) [46].

The relationship between road traffic noise and incident atrial fibrillation was also evaluated in a Danish prospective study, showing that an increase in noise levels per 10 dB was associated with a 6% (IRR: 1.06, 95% CI: 1.00–1.12) higher risk; however, this relationship was not independent of exposure to air pollution [47]. Since the growing demand for mobility is not only related to noise but also air pollution, it is important to differentiate between these two exposures in terms of evaluating health effects. However, a range of epidemiologic evidence supports the concept that both noise and air pollution independently contribute to disease development, while they also may interact with each other having additive negative effects [48, 49]. Importantly, exposure to air pollution was also shown to be independently associated with cerebrocardiovascular (e.g., stroke, ischemic heart disease, and hypertension) and psychological/psychotic disorders (e.g., depression, anxiety, and schizophrenia) and was found to share common pathophysiological pathways with noise exposure [50–53].

Furthermore, several studies have indicated substantial associations between traffic noise exposure and metabolic abnormalities, showing that higher exposure is associated with increased risk of obesity and diabetes mellitus [54–57], both well-established risk factors for cerebrocardiovascular [58, 59] and psychological disorders [60, 61].

### 3.6. Psychological Symptoms and Disorders

Another cross-sectional analysis based on data from the GHS including 15,010 subjects could demonstrate that depression and generalized anxiety disorder increased dose-dependently with the degree of total noise annoyance (highest annoyance rating of all analyzed categories of noise including aircraft, road traffic, and railway noise), even after adjustment for sex, age, and socioeconomic status [19]. Compared to no annoyance, the prevalence ratio (PR) for depression and generalized anxiety disorder increased steadily from slight, over moderate and strong to extreme annoyance. In case of extreme annoyance, the PR for depression was 1.97 (95% CI: 1.62–2.39) and for generalized anxiety disorder 2.14 (95% CI: 1.71–2.67) (Figure 3(b)). In addition, there are studies showing a positive association between aircraft noise exposure and the intake of tranquilizing and sleep-inducing drugs and antidepressants [62, 63].

These results have been confirmed in the prospective German Heinz Nixdorf Recall (HNR) study, showing that road traffic noise was associated with an increased RR of high depressive symptoms at follow-up of 1.29 (95% CI: 1.03–1.62 for >55 vs. ≤55 dB(A)) [64]. A further NORAH-based case-control study examined the association between exposure to traffic noise and risk of incident depression [20]. Road traffic noise was associated with an OR of 1.17 (95% CI: 1.10–1.25) for noise levels of >70 dB, an OR of 1.23 (95% CI: 1.19–1.28) was found for aircraft noise levels of ≥50 to <55 dB, and in case of railway noise, the OR was 1.15 (95% CI: 1.08–1.22) for noise levels of ≥60 to <65 dB. The highest OR of 1.42 (95% CI: 1.33–1.52) was revealed for a combined exposure to noise from all three sources at noise levels above 50 dB.

A small case-control study from Italy showed an increased OR for prevalent generalized anxiety disorder (OR: 2.0, 95% CI: 1.0–4.2) and anxiety disorder not otherwise specified (OR: 2.9, 95% CI: 1.0–4.1) among subjects exposed to aircraft noise [65]. Recently, two Dutch studies analyzed the influence of traffic noise on depression and anxiety [66, 67]. Leijssen et al. demonstrated that exposure to road traffic noise was associated with increased prevalent depressed mood (OR: 1.65, 95% CI: 1.10–2.48 for ≥70 vs. 45-54 dB(A)), independent of ethnic and socioeconomic inequalities between groups of exposure [66]. The case-control study by Generaal et al. indicated an OR of 1.26 (95% CI: 1.08–1.47) for depressive disorder and 1.29 (95% CI: 1.11–1.50) for anxiety disorder per 3.21 dB(A) increase in traffic noise levels [67].

Moreover, occupational noise has been linked to psychological symptoms. A Korean study found occupational noise annoyance to increase depressive symptoms and suicidal ideation in men and women [68]. Compared to no annoyance, the OR for depressive symptoms were 1.58 (95% CI: 1.12–2.23) in men and 1.49 (95% CI: 1.05–2.11) in women; for suicidal ideation, the corresponding OR were 1.76 (95% CI: 1.29–2.40) in men and 1.41 (95% CI: 1.01–1.97) in women. A study of Egyptian airport workers found an increased prevalence of symptoms of anxiety along with other somatic symptoms in noise exposed workers (34% vs. 18% in controls) [69].

## 4. Noise and Experimental Studies in Animals and Humans

Experimental data provide mechanistic pathways by which noise exposure may trigger pathophysiological alterations and contribute to disease development. However, evidence is limited and the molecular mechanisms underlying the relationship between noise exposure, mental stress, and risk for cerebrocardiovascular and psychological disorders are not completely understood, yet. Taken together, it has been proposed that autonomic perturbation and sympathoadrenal activation induced by chronic noise stress may lead to increased levels of circulating stress hormones and subsequent oxidative stress-induced endothelial dysfunction, an early predictor for atherosclerosis, accompanied by the release of proinflammatory mediators and activation of prothrombotic pathways [70, 71]. This concept is well supported by a recent human study linking emotional stress with increased risk of cardiovascular disease by demonstrating that increased amygdala activity, a brain region involved in stress, is associated with arterial inflammation (increased plaque burden) by ^18^F-fluorodexoyglucose PET/CT scanning technique [72]. Importantly, increased amygdala activity not only correlated with higher emotional stress burden but was also predictive for risk of future cardiovascular events (standardized HR: 1.59, 95% CI: 1.27–1.98), a finding that remained significant after multivariate adjustments.

### 4.1. Evidence from Human Studies

To date, only few studies of experimental, mechanistic nature have examined the underlying molecular mechanisms of noise effects in humans. The most comprehensive results in this context were achieved by Münzel and coworkers. In a field study of 75 healthy adults, Schmidt et al. evaluated the effects of simulated nighttime aircraft noise exposure on endothelial function as determined by flow-mediated vasodilation, sleep quality, and stress hormone levels [73]. Noise exposure was found to cause a dose-dependent worsening of endothelial function and sleep quality and to increase adrenaline levels proportionally, clearly in line with the implications of the “indirect pathway” of Babisch's noise reaction model. Of note, mental stress and depression per se were shown to contribute to endothelial dysfunction and atherosclerosis, while on the other hand, endothelial dysfunction and atherosclerosis may contribute to the incidence of depression via induction of hypothalamic-pituitary-adrenal axis overactivity, increased platelet activation, hypercoagulability, and activation of the inflammatory response [74, 75]. In addition, the pulse transit time was decreased, a parameter reflecting sympathetic arousal associated with elevated blood pressure, vascular tone, and stiffness. Interestingly, the worsening of endothelial function could be corrected by the single administration of the antioxidant vitamin C in a subgroup of subjects, which further suggests that an increased formation of reactive oxygen species and oxidative stress may be involved in the mediation of noise-induced endothelial dysfunction and cardiovascular complications.

A subsequent study by Schmidt et al. of 60 subjects with prevalent or with increased risk of ischemic heart disease found the above-described effects even more pronounced in this sample [76]. In addition to the previous findings regarding vascular function, sleep quality, and stress hormone levels, it has been shown that aircraft noise exposure is associated with increased systolic blood pressure (control group: 129.5 mmHg vs. noise group: 133.6 mmHg). Alongside these observations, further studies indicated that noise exposure causes autonomic imbalance by, e.g., increased heart rate, increased blood pressure, and sympathetic activation or parasympathetic withdrawal as well as increased arterial stiffness [77–81]. Overall, these results may provide key pathophysiological mechanisms by which noise induces adverse health effects as shown by the results of epidemiological studies on ischemic heart disease, arterial hypertension, stroke, myocardial infarction, arrhythmia, heart failure, depression, anxiety, and metabolic abnormalities.

In addition, Chang et al. showed that environmental noise exposure has differential impact on arterial compliance and resistance vessels. Whereas a 1-dB(A) increase in noise exposure was associated with an augmentation of 1.25 (95% CI: 1.10–1.42) %mL/mmHg in arterial compliance, a decrease of 2.12 (95% CI: -2.51 to -1.80) kdynes∗s/cm^5^ in arterial resistance was observed [82]. The same authors also demonstrated that road traffic noise exposure (≥ the median of noise levels) applied at specific frequencies may exert different impact on the risk for prevalent hypertension in 820 residents of central Taiwan with the 125 Hz frequency component displaying the most pronounced hypertensive effects (OR: 4.08, 95% CI: 1.57–10.63) [83]. Likewise, a retrospective cohort study identified the 4 kHz component of occupational noise as the most potent trigger of hypertension in 1,002 volunteers from 4 machinery and equipment manufacturing companies in Taiwan [84]. A 20 dB(A) increase in noise exposure at 4 kHz was associated with a 34% higher risk of hypertension (OR: 1.34, 95% CI: 1.01–1.77).

### 4.2. Evidence from Animal Studies

High noise levels (octave band noise: 80-100 dB, 8-16 kHz, 8 h/d for 20 d; 8 rats/group) increased plasma levels of stress hormones (corticosterone, adrenaline, noradrenaline, endothelin-1) and caused oxidative stress (increased malondialdehyde levels and decreased superoxide dismutase activity) in rats leading to an adverse cardiovascular phenotype as evidenced by severe endothelial dysfunction [85]. Other pathophysiological effects included higher mean arterial blood pressure and heart rate as well as higher levels of circulating nitrogen oxides (marker of inducible nitric oxide synthase activity) [85].

**Figure 4:**
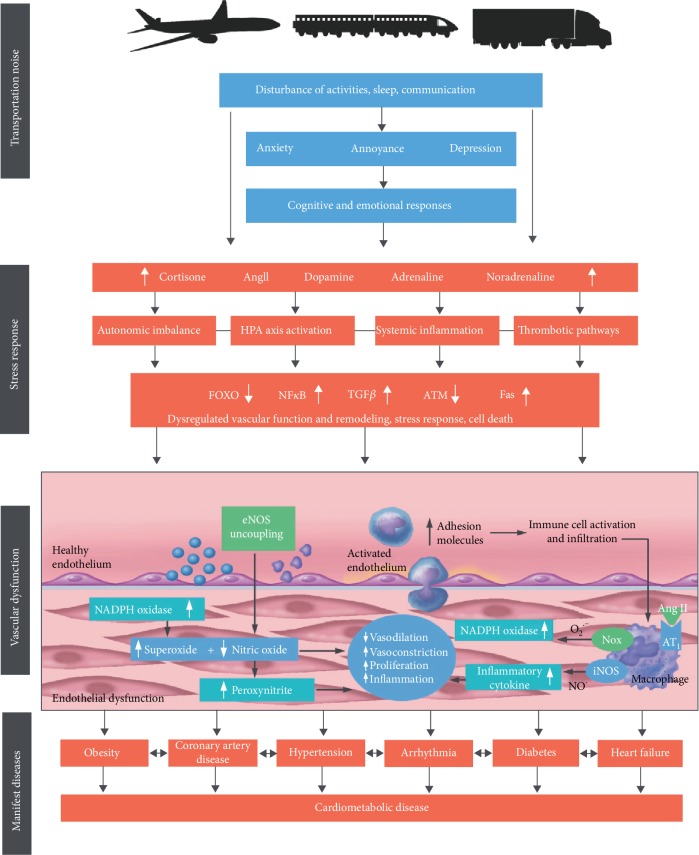
Noise causes annoyance and stress responses characterized by activation of the hypothalamic-pituitary-adrenal axis, oxidative stress-induced endothelial dysfunction, inflammation, thrombosis, and altered gene expression. Adapted from Münzel et al. [6]. Ang II: angiotensin II; AT_1_: angiotensin receptor type 1; ATM: ataxia telangiectasia mutated; eNOS: endothelial nitric oxide synthase; Fas: cell death signaling molecule (CD95); FOXO: forkhead-box-protein O3; HPA: hypothalamic-pituitary-adrenal; iNOS: inducible nitric oxide synthase; NADPH: nicotinamide adenine dinucleotide phosphate; Nox: NADPH oxidase; NO: nitric oxide; O_2_: oxygen; TGF: transforming growth factor. Adapted from Münzel et al. [6] with permission.

Recently, two comprehensive animal models in mice were established to study the molecular, nonauditory consequences of noise exposure. In our first study, mice were exposed to simulated aircraft noise for four days; in the control scenario, the mice were exposed to “white noise,” both conditions exhibiting the same noise intensity (i.e., 72 dB(A)) [86]. Aircraft noise exposure caused an increased stress response as indicated by increased levels of cortisol, noradrenaline, dopamine, angiotensin II, and endothelin-1. In addition to increased systolic blood pressure, increased expression of nicotinamide adenine dinucleotide phosphate (NADPH) oxidase isoform 2 (Nox2), uncoupling of endothelial nitric oxide synthase (eNOS), inflammation in the vasculature, and subsequent endothelial dysfunction were observed (Figure 4) [86]. The induction of oxidative stress and inflammatory pathways in the vasculature was indicated by increased levels of 3-nitrotyrosine-positive proteins, lipid peroxidation products, and interleukin-6 (IL-6) and a more pronounced infiltration of proinflammatory macrophages into the aortic wall, respectively [86]. The presence of the two radical-forming enzymes, Nox2 and uncoupled eNOS, led to subsequent decreased vascular bioavailability of NO and thus to endothelial dysfunction and high blood pressure. Moreover, next-generation sequencing (Illumina RNAseq) revealed downregulation of genes encoding for antioxidant systems (e.g., intracellular SOD1, glutathione peroxidase-1, forkhead-box-protein O3), while an upregulation of proapoptotic factors for enhanced cell death (e.g., caspases, Fas, p38) was observed [86]. Interestingly, these effects were not seen in the control scenario with white noise, implicating that the stress-inducing character of aircraft noise exposure rather than noise exposure per se is crucial in determining adverse effects.

In our second study, aircraft noise exposure was furthermore found to induce cerebral oxidative stress and neuroinflammation, which was associated with a dysregulation of genes encoding the circadian clock, all of which caused systemic inflammation (e.g., increased expression of inducible NO synthase), oxidative stress, and endothelial dysfunction [87]. Of note, uncoupling and downregulation of neuronal NO synthase was observed, which will ultimately lead to impaired neuronal NO signaling and dysregulation of this important neuronal signaling molecule may explain, at least in part, the cognitive impairment in school children in response to noise [88]. Interestingly, noise-induced cerebral and vascular effects were present when mice were exposed during sleep but not awake phase, suggesting nighttime interference and impaired sleep quality leading to more stress as a crucial step. Genetic deletion of Nox2 not only prevented cardiovascular but also cerebral complications inflicted by noise clearly identifying oxidative stress as a major component in noise-triggered cerebrocardiovascular risk.

In summary, these findings indicate oxidative stress-induced endothelial dysfunction in response to noise exposure-related mental stress as key factors in the relationship between cerebrocardiovascular and psychological disorders. Importantly, environmental noise exposure appears to share common pathophysiological pathways with traditional cerebrocardiovascular risk factors such as obesity, diabetes mellitus, hypertension, and smoking with the main consequence of oxidative stress and endothelial dysfunction. However, as discussed above in Section 4.1 (last paragraph), specific frequency components of traffic noise exert differentially pronounced health effects in human subjects [83, 84] and, accordingly, translation of animal experimental data may be hampered by species differences in noise perception. Moreover, we have shown that white noise exposure, despite application of similar sound pressure levels and despite presence of a continuous band of frequencies in white (or pink) noise, displayed no adverse cardiovascular effects in mice [86], suggesting that the noise pattern (e.g., crescendo and diminuendo character, tantamount to increasing and decreasing intensity/loudness, of aircraft noise as well as particular breaks) may be of importance as well.

### 4.3. Oxidative Stress and Inflammation as Common Features of Psychological Disorders

The above-described mechanisms of noise-induced cerebral oxidative stress may also contribute to the onset of psychological disorders in response to chronic noise. Obviously, the origin of the mental stress plays a minor role for the activation of these stress response pathways, which will all converge at the level of oxidative stress and inflammation. Mice that were subjected to daily restraint and cage-switch stress for one week developed severe inflammation and hypertension [89, 90].

Likewise, human data also support this concept as a meta-analysis (23 studies, 4,980 subjects) revealed a 0.55 of 1 standard deviation increase in oxidative stress markers among individuals with depression compared with those without depression, which was further supported by a negative correlation between depression and antioxidant status [91]. Another meta-analysis (10 studies, 1,308 subjects) found that oxidative stress markers 8-hydroxy-2′-deoxyguanosine and F2-isoprostanes are increased in depression [92]. Qualitatively, another meta-analysis came to the same conclusion (29 studies, 3,961 subjects) and antidepressant therapy restored levels of oxidative stress markers and antioxidants [93]. A report on 96,989 individuals from two independent cohort studies revealed that higher plasma levels of the physiological antioxidant uric acid are associated with lower risk of depression hospitalization and lower antidepressant medication use [94]. Likewise, combining several inflammatory biomarkers (e.g., C-reactive protein, IL-6, tumor necrosis factor alpha) within a meta-analysis (53 studies, 2,467 cases and 2,360 controls) could differentiate individuals with bipolar disorder from healthy controls and indicate a specific mood-phase signature [95].

The impact of mental stress on oxidative stress pathways and inflammation was reviewed in full detail by Siegrist and Sies [96] as well as in two recent articles within the forum issue “Oxidative stress and redox signaling induced by the environmental risk factors mental stress, noise and air pollution” [97, 98]. Another review article put forward the concept that severe life stress is associated with cerebral oxidative stress with Nox2 as a major source [99].

## 5. Conclusions

**Figure 5:**
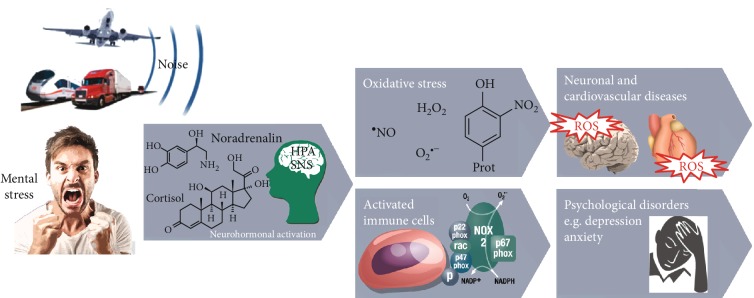
Environmental noise exposure and subsequent mental stress cause a stress reaction through activation of either the hypothalamus-pituitary-adrenal (HPA) axis with subsequent cortisol release or by the activation of the sympathetic nervous system (SNS) with subsequent catecholamine formation. As a consequence, cerebral and vascular inflammatory and oxidative stress pathways as well as altered gene expression become active, leading to endothelial dysfunction. Taken together, these consequences contribute and interact with traditional risk factors, leading to neuronal, cerebrocardiovascular, and psychological disorders. Modified from Münzel et al. [100] and adapted from Daiber et al. [71] with permission.

In summary, the present review elucidates important mechanisms by which environmental noise exposure induces cerebrocardiovascular and psychological disorders (Figure 5). Noise interferes with communication, disturbs daily activities, and disrupts sleep, leading to mental stress. Upon chronic exposure, stress responses as evident by increased stress hormone levels lead to autonomic imbalance, oxidative stress, inflammation, and endothelial dysfunction, which then accelerates the development of cerebrocardiovascular risk factors and disease. Importantly, since noise exposure reflects mental stress, it favors the onset of psychological symptoms and disorders, which in turn is associated with cerebrocardiovascular dysfunction, highlighting the interrelationship between mental stress/psychological disorders and cerebrocardiovascular disease. Further studies, in particular with assessment of noise-induced cerebrocardiovascular and psychological consequences in context of one another, are warranted to gain more insight in the mechanisms underlying this relationship. In conclusion, environmental noise has to be acknowledged as an important risk factor for cerebrocardiovascular and psychological health, which has to be mentioned in corresponding current guidelines.
